# Nucleoredoxin Knockdown in SH-SY5Y Cells Promotes Cell Renewal

**DOI:** 10.3390/antiox10030449

**Published:** 2021-03-13

**Authors:** Lucie Valek, Irmgard Tegeder

**Affiliations:** Institute of Clinical Pharmacology, Medical Faculty, Goethe-University, 60590 Frankfurt, Germany; valek@med.uni-frankfurt.de

**Keywords:** nucleoredoxin, SH-SY5Y neuroblastoma cells, hydrogen peroxide, cell cycle, autophagy, chaperone-mediated autophagy, ubiquitin ligase

## Abstract

Nucleoredoxin (NXN) is a redox regulator of Disheveled and thereby of WNT signaling. Deficiency in mice leads to cranial dysmorphisms and defects of heart, brain, and bone, suggesting defects of cell fate determination. We used shRNA-mediated knockdown of NXN in SH-SY5Y neuroblastoma cells to study its impact on neuronal cells. We expected that shNXN cells would easily succumb to redox stress, but there were no differences in viability on stimulation with hydrogen peroxide. Instead, the proliferation of naïve shNXN cells was increased with a higher rate of mitotic cells in cell cycle analyses. In addition, basal respiratory rates were higher, whereas the relative change in oxygen consumption upon mitochondrial stressors was similar to control cells. shNXN cells had an increased expression of redox-sensitive heat shock proteins, Hsc70/HSPA8 and HSP90, and autophagy markers suggested an increase in autophagosome formation upon stimulation with bafilomycin and higher flux under low dose rapamycin. A high rate of self-renewal, autophagy, and upregulation of redox-sensitive chaperones appears to be an attractive anti-aging combination if it were to occur in neurons in vivo for which SH-SY5Y cells are a model.

## 1. Introduction

High levels of reactive oxygen species (ROS) such as hydrogen peroxide (H_2_O_2_) are harmful and lead to irreversible protein oxidation and serious damage to DNA, lipids, and matrix components [1], but low amounts of reactive oxygen species act as signaling molecules in cellular pathways by modifying cysteine residues in redox-sensitive proteins, changing thereby enzymatic activity or protein–protein and protein–nucleotide interactions [2]. To maintain homeostatic levels and protect proteins against oxidative damage, cells are equipped with an antioxidant system, including superoxide dismutase and several redoxins of the thioredoxin, peroxiredoxin, and glutaredoxin families [3,4].

Nucleoredoxin (NXN) is an oxidoreductase of the thioredoxin family. It was originally detected in the nucleus, leading to its name, but was later localized predominantly in the cytoplasm [5,6]. NXN contains three thioredoxin-like domains, the central one comprising a catalytically active WCPPC (Trp, Cys, Pro, Pro, Cys) motif that is involved in the reduction of disulfide bonds in target proteins [5]. In contrast to the expected function of a typical redoxin, a recent study found a high proportion of reduced proteins in NXN-depleted SH-SY5Y neuroblastoma cells, suggesting that oxidase-like activity of NXN predominates in these cells [7]. In addition to a contribution to global redox balancing [7], NXN specifically modulates WNT signaling via a redox-dependent interaction with the WNT inhibitor, disheveled (Dvl) [8,9]. The interaction was strengthened in reducing conditions, resulting in an inhibition of WNT/-catenin signaling [8] and hence, cell proliferation and cell fate determination [10]. WNT signaling is crucial for cell renewal and differentiation. Oppositely, the interaction was weakened in oxidizing environments [8]. Irrespective of the oxidative state, NXN retained a pool of inactive Dvl by preventing its ubiquitination and subsequent proteasomal degradation [11]. Dvl and -catenin are degraded via the proteasome and via autophagy, which are important negative feedback control mechanisms of WNT signaling [12]. Both pathways are controlled via redox modifications of candidate pathway proteins [13,14], but NXN-mediated regulations of protein homeostasis have not yet been studied.

Proteomic studies using SH-SY5Y cells with an active site Cys-mutated NXN suggested broad implications of NXN in metabolism and morphogenesis [7], the latter in agreement with WNT signaling. It is important to note that an N-ethyl-N-nitrosourea (ENU) mutation screen in mice revealed a splice site mutation of NXN in mice [15], suggesting NXN mutations in the context of genetic diseases. In humans, a mutation of NXN has indeed been associated with a recessive form of Robinow disease, which is a rare genetic disorder mainly leading to bone deformities. The studies point to functions of NXN that go beyond its redox activities. Interaction screens found further partners of NXN in addition to Dvl including histone deacetylase 6 (HDAC6) [16] and heat shock protein of 90 kilodalton (HSP90) [7]. They point to autophagy pathways and suggest that NXN might help to maintain the redox state-dependent digestibility of proteins and protein aggregates [14,17,18].

HDAC6 recruits and transports misfolded polyubiquitinated proteins to aggresomes/autophagosomes for a subsequent degradation by lysosomes [19], whereas HSP90 maintains the stability of autophagy proteins such as Beclin [20]. Importantly, both of these candidates are subject of redox-guided regulation through modification of critical cysteines [21,22]. In addition, we have previously shown that chaperone-mediated autophagy (CMA) is regulated via redox modification of Cys17 of HSPA8/HSC70, which negatively determines its ATPase activity, chaperoning functions, and clathrin-mediated endocytosis [13], suggesting that NXN may affect the degradation of CMA target proteins and lipid droplets, which are degraded via CMA [23]. Since NXN mainly acts as oxidoreductase on protein thiols [7], it likely helps to maintain a balanced oxidative state of candidate proteins involved in developmental processes like WNT signaling and protein homeostasis.

To gain further insight into the functions of NXN for cell renewal and proteostasis, we have created stable NXN knockdown (shNXN) and scrambled (scrNXN) control SH-SY5Y human neuroblastoma cell lines and assessed their proliferation, cell cycle distributions, resistance to redox stress, cell respiration, and adaptation of redox-sensitive autophagy.

## 2. Materials and Methods

### 2.1. Cell Culture

Human neuroblastoma cells (SH-SY5Y) were grown in 1:1 EMEM/F12 medium supplemented with heat-inactivated 15% fetal bovine serum, 100 U/mL penicillin/streptomycin, 1× Non-Essential Amino Acid, and 2 mM glutamine at 37 °C in 5% CO_2_ atmosphere at 37 °C in a humidified tissue culture incubator.

### 2.2. Nucleoredoxin Knockdown (shNXN) Neuroblastoma Cells

Stable cell lines of NXN knockdown SH-SY5Y cells (shNXN) and of scrambled shRNA control cells were produced by lentiviral-mediated transduction of shRNA against NXN using the lentiviral plasmid vectorpLenti-GFP-shNXN-A, -B, -C (pool of sh-A, sh-B, and sh-C). Control cells were transduced with the lentivirus carrying scrambled short hairpin RNAs (pLenti-GFP-scrNXN). Lentiviral particles were produced by transient co-transfection of HEK293T cells with 10 µg of vector DNA, together with three helper plasmids (5 µg of pMDL/RRE, 3 µg of RSV-Rev, and pMD2G-VSVG) using the calcium phosphate precipitation method. Cell supernatants containing lentivirus particles were harvested 48 h after transfection, passed through a 0.45 µm filter, and concentrated by centrifugation for 90 min at 40,000 rpm at 4 °C. The virus pellet was suspended in 25 µL cold 1× PBS and the titers were adjusted to 2–3 × 10^7^ transduction units/mL. SH-SY5Y cells were transduced at 10 MOI in complete medium. After 3 days, the efficiency of virus infection of pGFP-C-shLenti was determined by flow cytometry of GFP expression. GFP-positive cells were sorted by FACS (FACS-Aria Cell sorter, Becton Dickinson, Germany) and antibiotic (Puromycin) selection.

### 2.3. Modulation of Autophagy and of Redox Stress

ScrNXN and shNXN SH-SY5Y cells (1 × 10^6^) were grown to 60% confluence and stimulated with 1 µM bafilomycin A1, 0.1 µM or 1 µM rapamycin (1 mM stock in ethanol or DMSO) to assess autophagy. The drugs were added to the culture medium. An equal volume of vehicle was added to the control cells.

To expose cells to oxidative stress, cells were stimulated with increasing concentration of hydrogen peroxide ranging from 0 to 200 µM H_2_O_2_ (hydrogen peroxide 30%, ROTH) for 24 h. The concentration range of H_2_O_2_ was based on previous studies in SH-SY5Y cells, which mostly used a range of 100–800 µM H_2_O_2_ [13,24,25,26,27,28].

### 2.4. Cell Cycle Analysis Using Flow Cytometry

ScrNXN and shNXN cells were seeded in 10 cm dishes, starved in serum-free medium for 3 days, and treated with 100 µM H_2_O_2_ or vehicle for 24 h. Cells were harvested by trypsinization, fixed with 80% ethanol, washed, incubated for 5 min with 0.125% Triton-X-100, washed again, and stained with propidium iodide in PBS containing 0.2 mg/mL RNAse A. Stained cells were analyzed by flow cytometry (FACS Canto II, Becton Dickinson, 69126 Heidelberg, Germany). The cell cycle distribution, i.e., the percentage of cells in subG1, G0/G1 (2N), S and G2/M (4N) phase, was assessed using FLowJo V10.6 using the univariate algorithm, which is similar to the Watson Pragmatic algorithm [29]. The model assumes Gaussian distributions of the 2N and 4N populations (G0/G1 and G2/M) and then uses a subtractive function to reveal the S-phase population. The cells within one standard deviation of the 2N and 4N medians are subtracted from the data, and the remaining cells (S-phase) are fit to a polynomial function, which is convoluted with the Gaussian distributions to form the complete model.

### 2.5. WST Assay

Cell proliferation was determined based on the metabolic activity of mitochondrial dehydrogenases using a WST-1 assay (Roche, 68305 Mannheim, Germany). In viable cells, the mitochondrial succinate tetrazolium dehydrogenase system catalyzes the conversion of the red tetrazolium salt WST-1 (4-[3-(4-Iodophenyl)-2-(4-nitrophenyl)-2H-5-tetrazolio]-1,3-benzole-disulfonate) to dark red formazan, which is analyzed photometrically. In brief, cells were seeded in a concentration of 2 × 10^3^ cells/well of a 96-well plate, cultivated for 24 h, and stimulated with different concentration of H_2_O_2_ (0–200 µM) for 24 h in quadruplets. Ten microliters of WST-1 were added and cells were incubated for an additional 4 h. Optical density (OD) at 440 nm was determined relative to the OD at 650 nm using an ELISA multiplate reader (Tecan Infinite 200 Pro). The background was adjusted using 100 μL medium with 10% WST-1 reagent without cells.

### 2.6. Colony Formation Assay

SH-SY5Y cells (1000 cells per plate) were seeded in 6 cm dishes and allowed to form colonies. After seven days, colonies were washed with PBS, fixed with ice-cold methanol for 3 min, and stained with crystal violet (0.5% (*w*/*v*) crystal violet in 25% (*v*/*v*) methanol) for 10 min. Excess crystal violet was washed off with distilled water and stained cells were analyzed by light microscopy. Tiled images were captured to grab multiple sites of the culture. After background subtraction and auto-threshold setting, cells and colonies were counted in FIJI ImageJ with the “Particle Counter”. Stacks and image montages were created in ImageJ.

### 2.7. Immunofluorescence Analysis of Nucleoredoxin and Cell Proliferation

Cell cultures were washed in 1× PBS, fixed in 4% PFA and immunostained. Cells were permeabilized in 1× PBS with 0.1% Triton-X-100 (PBST), then blocked with 5% bovine serum albumin (BSA)/1% normal goat serum (NGS) in PBST (3% BSA/1% NGS/PBST), and subsequently, incubated overnight with the first antibodies in 1% BSA/1% NGS/PBST at 4 °C. After washing three times with 1× PBS, slides were incubated with secondary antibodies for 2 h at room temperature, followed by 10 min incubation with DAPI and embedding in Aqua-Poly/Mount. Blocking solutions were adjusted if necessary. Primary antibodies included nucleoredoxin, Ki67, the mitochondrial membrane markers TOM20, cytochrome oxidase, COX-IV, dynamin-related protein 1 (Drp1) and cytoskeleton markers, vimentin and phalloidin (Supplementary Table S1). Secondary antibodies were labeled with fluorochromes (Invitrogen). For live cell labeling with wheat germ agglutinin (WGA), cells were incubated with 1 µg/mL WGA-AF488 in culture medium for 10 min at 37 °C, washed 2× in 1× PBS, fixed in 4% PFA for 15 min at 37 °C, and then immunostained as above.

Images were captured on an inverted fluorescence microscope (BZ-9000 KEYENCE, Germany, and Axio Imager Z1, Zeiss, Germany). FIJI ImageJ was used for counting Ki67-positive proliferating cells with the particle counter implemented in ImageJ after threshold setting and generation of binary images. Nuclei were counter-stained with DAPI. Mitochondrial shape was estimated by analyzing the sphericity in ImageJ.

### 2.8. Western Blot

Whole cell protein extracts were prepared in PhosphoSafe Buffer (Sigma Aldrich, Darmstadt, Germany) or RIPA lysis buffer (Sigma Aldrich) containing a protease inhibitor cocktail (Roche) and PMSF 10 µg/mL, separated on a 10% SDS-PAGE gel (30 µg/lane), transferred to nitrocellulose membranes (Amersham Pharmacia Biotech, 79111 Freiburg, Germany) by wet-blotting or electro-blotting. Blots were blocked and developed in Odyssey buffer or 5% skim milk in 1× PBS/Tween 20. For detection of specific proteins, blots were incubated with primary antibodies (see Supplementary Table S1). Glyceraldehyde 3-phosphate dehydrogenase (GAPDH) was used as loading control. Secondary antibodies were conjugated with IRDye 680 or 800 (1:10,000; LI-COR Biosciences, 61352 Bad Homburg, Germany) and blots were analyzed on the Odyssey Infrared Imaging System (LI-COR Biosciences). The ratio of the respective protein band to the loading control was used for semiquantitative analysis (Image Studio Light^®^, Odyssey).

### 2.9. Seahorse Analysis of Oxygen Consumption and Extracellular Acidification

Mitochondrial respiration and extracellular acidification were analyzed simultaneously on a Seahorse XFp instrument using an XFp “Cell Mito Stress Test” (Agilent Technologies, 76337 Waldbronn, Germany) [30,31,32,33,34,35,36,37]. This is a plate-based live cell assay for monitoring of oxygen consumption and extracellular acidification rates in live cells. The Seahorse extracellular flux analyzer continuously measures oxygen concentrations and proton flux in the cell supernatant over time, and these measurements are converted in OCR and ECAR values and enable a direct quantification of mitochondrial respiration and glycolysis, respectively. Extracellular acidification is caused mainly by lactate through anaerobic glycolysis. Depending on the conditions, ECAR is contributed by protons produced from carbon dioxide that is generated in the citric acid cycle during respiration [38]. This contribution is eliminated by blocking oxidative phosphorylation with, e.g., oligomycin.

One day before the analyses, shNXN SH-SY5Y cells and control cells (scrNXN or wildtype) were plated at 15,000 or 20,000 cells per well of a Seahorse culture miniplate to reach 80–90% cell density for analysis. One hour before the start of the analysis, the culture medium was exchanged with Seahorse XF medium consisting of DMEM + 1 mM pyruvate + 2 mM glutamine + 10 mM glucose to allow for analysis of basal respiration under full nutrient supply. Subsequently, oligomycin (1.5 µM) was added to assess ATP generation, proton leakage and extracellular acidification. Oligomycin blocks proton flow through ATP synthase, blocking thereby ATP-linked oxygen consumption, which leads to a drop in OCR and rise in ECAR. Subsequently, FCCP (carbonyl cyanide-4-(trifluoromethoxy)phenylhydrazone; 1.0 µM) was injected to assess maximum oxygen consumption. FCCP transports H^+^ across the mitochondrial membrane, leading to a collapse of membrane potential and rapid consumption of oxygen. It is used to initiate maximal respiration. Finally, residual non-mitochondrial respiration was determined after inhibition of complex I with rotenone (0.5 µM) and complex III with antimycin A (0.5 µM).

Cells were then trypsinized with 20 µL of 1% Triton-X-100/PBS for 5 min, and 5 µL of cell lysates were mixed 1:1 with distilled water for protein analysis using Bradford reagent. Oxygen consumption rate (OCR) and extracellular acidification rate (ECAR) were normalized according to the protein content, and OCR and ECAR are expressed as percentage change versus basal respiration to allow for a combined analysis of separate runs. Data analysis was performed using the Seahorse XF Wave^®^ software, Version 2.6.

### 2.10. Data analysis and Statistics

GraphPad Prism 8.4 and 9.0 were used for statistical evaluation. Data are presented as means ± SD. Sample sizes are given in the respective figure legend. Data were analyzed using univariate or 2-way analysis of variance (ANOVA) and subsequent post hoc *t*-tests versus scrNXN cells or versus baseline conditions with an adjustment of alpha according to Dunnett, or group-wise according to Šidák. Western blot results were compared by non-parametric Wilcoxon matched pairs rank test. *p* was set at 0.05 for all statistical comparisons.

## 3. Results

### 3.1. Higher Proliferation Rate in shNXN Knockdown Cells

**Figure 1:**
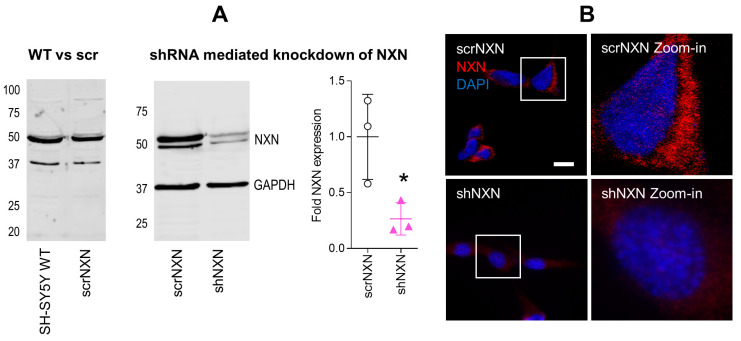
Expression of nucleoredoxin in NXN knockdown SH-SY5Y neuroblastoma cells. (**A**) Exemplary Western Blot and quantification of NXN (49 kDa) in wildtype SH-SY5Y human neuroblastoma cells and in stably transduced scrNXN and shNXN SH-SY5Y cells. Cells were transduced with a lentiviral vector carrying small hairpin RNAs against NXN (shNXN) or scrambled shRNA (scrNXN). GAPDH (37 kDa) was used as loading control. The data show the mean + SD of 3 experiments, and they were compared by Student’s *t*-test. The asterisk shows a significant result, * *p* < 0.05. (**B**) Immunofluorescence analysis of NXN with nuclear DAPI counterstain in scrNXN and shNXN SH-SY5Y cells. Scale bar: 20 µm.

We generated SH-SY5Y cells with stable shRNA-mediated knockdown of NXN. Scrambled shRNA transduced cells (scrNXN) were used as controls. The expected expression was confirmed by Western blot (Figure 1A) and immunofluorescence (Figure 1B). The expression of NXN in shNXN cells was reduced to about 20–25% of the scrNXN control cells in Western blots and the immunofluorescence signal of NXN was not detectable in shNXN cells.

**Figure 2:**
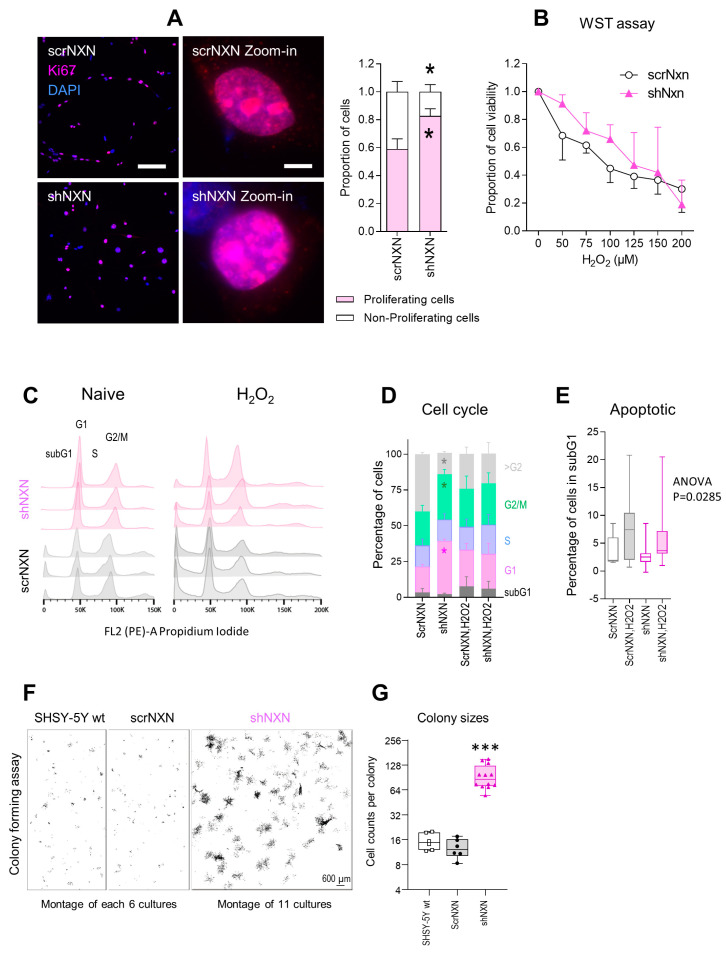
Proliferation and cell cycle analysis of shNXN knockdown SH-SY5Y cells. (**A**) Exemplary immunofluorescence images of the proliferation marker Ki67 and its quantification. The inserts show zoom-in images of individual nuclei. Asterisks show significant differences between groups; * *p* < 0.05. Scale bars: 50 µm, 5 µm for inserts. Sample sizes *n* = 5–7 cultures per group. (**B**) WST assay showing the viability of scrNXN and shNXN SH-SY5Y cells after stimulation with 0–200 µM H_2_O_2_. The WST assay measures the metabolic activity of viable cells. Sample size *n* = 6 per group and time point. Two-way ANOVA not significant. (**C**,**D**) Flow cytometry analysis of the cell cycle distributions, based on propidium iodide staining of the DNA content in vehicle and H_2_O_2_ (100 µM) stimulated cells. C shows exemplary histograms. The stacked bar charts in D show the relative frequency of cells in G1 (N2), S and G2/M (N4). Data show the means and SD of 6 independent cultures per group; 1 x exp5 cells were counted. Exemplary curve fitting according to the Watson Pragmatic algorithm are in Supplementary Figure S1. shNXN showed higher frequencies of cells in G1 and G2/M, suggesting a higher fraction of proliferating cells. (**E**) The box plots show the percentages of cells in the subG1 phase, which are considered apoptotic/dying cells. The box is the semi-interquartile range, and the line is the median. Sample size *n* = 6. Two-way ANOVA *p* = 0.0285, post hoc n.s. (**F**,**G**) Exemplary images and quantification of cell colonies after seeding of single cells in a colony formation assay. The images show montages of 6 or 11 cell culture images. The size of the colonies is revealed as number of cells per colony. Data were compared with one-way ANOVA and subsequent post hoc analysis according to Šidák. *** *p* < 0.001.

Cell proliferation and viability were assessed by Ki67 immunofluorescence and WST assays, respectively. Counts of cells with nuclear Ki67 revealed a higher fraction of actively proliferating cells in shNXN cultures (Figure 2A). Both groups responded equally towards stimulation with hydrogen peroxide with a drop of metabolic activity as assessed by WST assays. Viability declined over time without differences between groups (Figure 2B).

Cell cycle analyses of naïve cells and cells exposed to H_2_O_2_ (Figure 2C–E) showed that shNXN cells had higher G1 and G2/M peaks, and the histograms were shifted to the right, suggesting a higher rate of cell cycle reentry and a lower fraction of polyploid/polynucleated cells. Statistically, two-way ANOVA showed a highly significant interaction of “group X cell cycle phase” between shNXN and scrNXN for G1, G2/M (both increased in shNXN) and >G2 (reduced in shNXN) (*p* < 0.0001). The data suggested a higher proliferation of shNXN cells.

We used a colony formation assay to further assess cell renewal and colony formation (Figure 2F,G). shNXN cells proliferated much faster than scrNXN or wildtype SH-SY5Y cells, which were alike. The sizes of the colonies were about 10-fold higher in shNXN cultures compared with the other two. The proliferation advantage requires higher energy.

### 3.2. Higher Basal Respiratory Rate but Similar Stimulus Response in shNXN Cells

**Figure 3:**
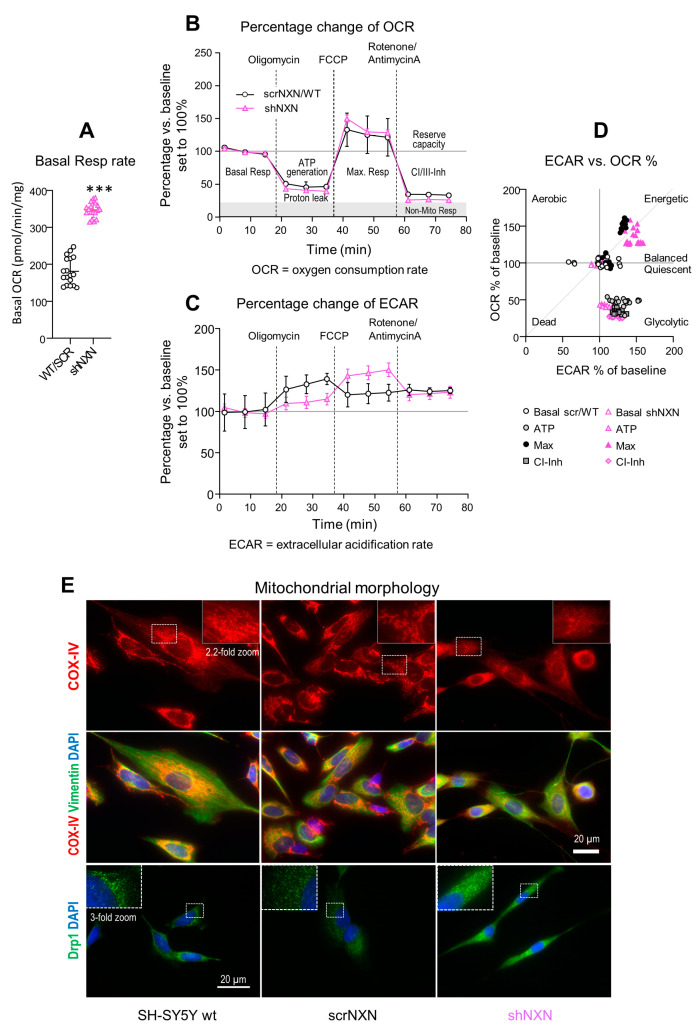
Seahorse analysis of oxidative respiration and extracellular acidification. (**A**) Mitochondrial respiration was analyzed on the Seahorse analyzer. The oxygen consumption rate (OCR) shows the absolute basal respiration (every 3 time points per culture) in shNXN and scrNXN/wildtype SH-SY5Y cells in the presence of full nutrient supplements (pyruvate, glutamine and glucose). Basal respiration differed significantly between groups. Sample size *n* = 6 cultures per group, *** unpaired, 2-tailed *t*-test *p* < 0.001. (**B**,**C**) Oxygen consumption (A: OCR) and extracellular acidification (B: ECAR) in the “substrate-uncoupler-inhibitor-titration” protocol measured OCR and ECAR upon mitochondrial stressors (Cell Mito Stress Test). After basal respiration (in the presence of pyruvate, glutamine and glutamate), oligomycin was applied to assess ATP generation and proton leakage, subsequently FCCP to assess maximum respiration, and finally rotenone (Complex-I inhibitor) plus antimycin A (Complex-III inhibitor) to assess residual non-mitochondrial respiration. Oxygen consumption (A: OCR) and extracellular acidification (B: ECAR) were normalized according to the protein content, and OCR and ECAR are expressed as percentage change versus basal respiration. Sample sizes were *n* = 6 per group. scrNXN and wildtype SH-SY5Y were pooled. Time courses were compared by 2-way ANOVA for “group” X “stimulus”. There were no significant differences between groups for OCR, but ECAR showed a significant interaction, *p* < 0.05. (**D**) The ECAR versus OCR plot shows the energy map and clustering according to the stimulus, but no significant differences between groups. At maximum respiration, control cells split in two clusters, whereas all shNXN cells were highly energetic. (**E**) Morphology of mitochondria in wildtype SH-SY5Y, scrNXN and shNXN cells immunostained with anti-cytochrome oxidase IV (COX-IV) or dynamin-related protein 1 (Drp1). Vimentin shows the cytoskeleton and DAPI the nuclei. The dotted rectangles show the regions which were used for 2.2-fold zoom-ins. shNXN mitochondria display as small-dispersed dots, whereas mitochondria show typical filamentous-net morphology in wildtype and scrambled control cells.

Highly proliferating cells need more ATP. They may increase the respiratory rate or glycolysis to meet the requirements. We therefore assessed mitochondrial respiration, ATP generation, and the balancing of oxygen consumption (OCR, Figure 3A,B,D) and extracellular acidification rate (ECAR, Figure 3C,D), key indicators of mitochondrial respiration and glycolysis, respectively [30,31,32,33,34,35,36,37,39]. Indeed, absolute basal OCR in the presence of full nutrients and supplements (pyruvate, glutamine and glucose) was much higher in shNXN cultures (Figure 3A). OCR and ECAR levels were therefore normalized to the respective baseline levels to compare the response to mitochondrial blockers between groups (Figure 3B,C).

OCR decreased upon injection of the ATPase inhibitor, oligomycin, in both groups (Figure 3B), and the relative drop of ATP-linked OCR was equal. In both groups, OCR rose to the maximum after injection of FCCP that disrupts the mitochondrial membrane potential (Figure 3B). Finally, the injection of rotenone/antimycin A blocked Complex-I/III mitochondrial respiration and showed the residual non-mitochondrial respiration rate. Again, the response was equal in both groups (Figure 3B).

The time course of the extracellular acidification rate, ECAR, revealed stimulus-dependent differences between groups (Figure 3C) (ANOVA *p* < 0.0001 for the interaction “group X stimulus”). Acidification of control cells was strongest under oligomycin, whereas acidification in shNXN reached the maximum only after FCCP, i.e., during maximum oxygen consumption, suggesting a higher ATP demand of shNXN cells [38].

The OCR versus ECAR plot (Figure 3D) revealed similar stimulus-dependent energetic states. There were three clusters showing energetic cells (OCR and ECAR 150%), balanced/quiescent cells (OCR and ECAR 100%) and glycolytic cells (ECAR > OCR). The clustering was similar in both groups, but during FCCP (maximum respiration), all shNXN cells were highly energetic, whereas this was true only for half of the control cells.

The higher rate of basal respiration plus differences of stimulus-dependent ECAR curves agree with a higher rate of proliferation of shNXN SH-SY5Y cells. The maximum ECAR under oligomycin normally reflects the glycolytic capacity in most cells, but in highly energetic cells, maximum ECAR is reached only upon further stimulation of oxygen consumption and ATP demand, here with FCCP [38].

We performed immunofluorescence studies of mitochondria (COX-IV, Drp1) and cytoskeleton (vimentin) to see if the differences in mitochondrial respiration were reflected by differences in morphology. Drp1 is a marker of mitochondrial fission. Control cells (wildtype and scrNXN) had typical perinuclear well-organized nets of filamentous mitochondria whereas mitochondria in shNXN were dot-like, more dispersed and gave a frosted glass impression. The morphologic differences agree with the high rate of cell renewal of shNXN cells, because previous studies show that mitochondrial fusion and lengthening occur during cell maturation and aging, whereas mitochondrial fission is high in fast proliferating cells [40,41,42,43].

### 3.3. Higher Expression of Redox Sensitive Heat Shock Proteins and Enhanced Autophagy

**Figure 4:**
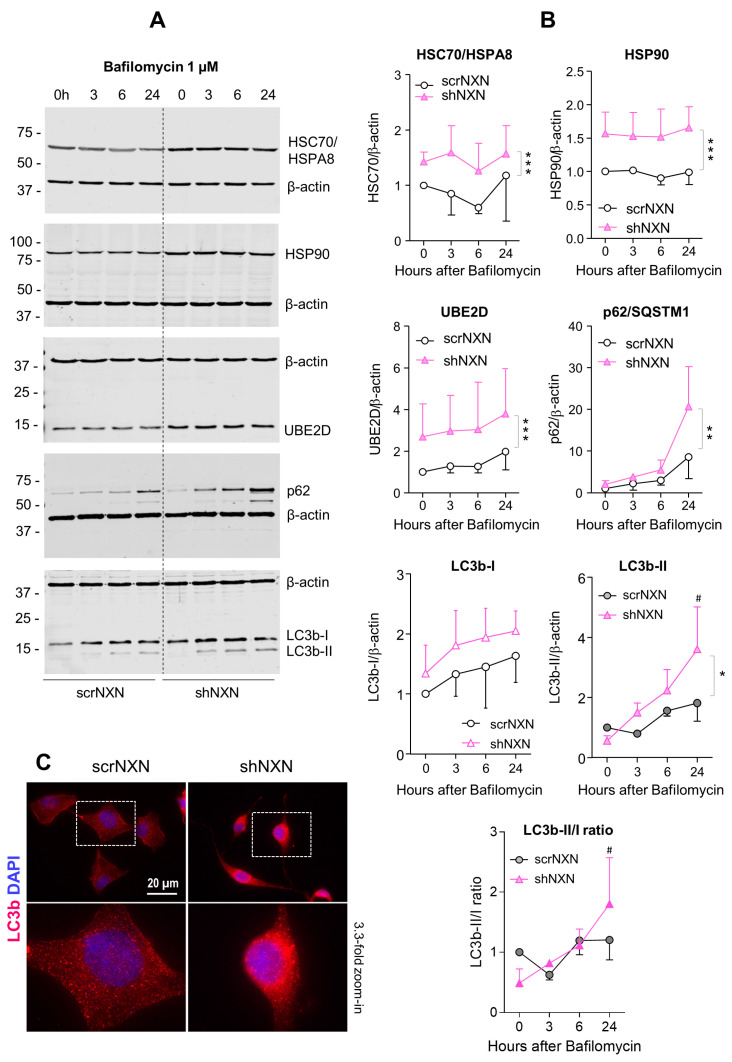
Western blot analysis of autophagy markers upon bafilomycin stimulation. (**A**,**B**) scrNXN and shNXN neuroblastoma cells were stimulated with 1 µM bafilomycin to block the lysosomal acidification and autophagolysosomal cargo breakdown. Exemplary blots and quantification show the time courses of heat shock cognate, HSC70/HSPA8, heat shock protein, HSP90, ubiquitin 2 ligase (UBE2D), sequestosome 1, SQSTM1/p62 and LC3b-I and LC3b-II, and the LC3b-II/I ratio. Blots were sequentially developed, and infrared images superimposed. Intensities were normalized for blot loading and show the mean + SD of 3 experiments. Statistical comparison by Wilcoxon matched pairs rank test. Asterisks indicate differences between groups. * *p* < 0.05; ** *p* < 0.01, *** *p* < 0.001. The # shows a significant increase from baseline. Raw Western blot data in Supplementary Figures S2 and S3. (**C**) Immunofluorescence analysis of LC3b (LC3-I and LC3b-II) in naïve scrNXN and shNXN SH-Sy5Y cells. The scale bar is 20 µM. The dashed rectangle reveals the area, which was used for 3.3-fold zoom-in of the bottom panel. Please note the different morphology of the faster proliferating shNXN cells.

**Figure 5:**
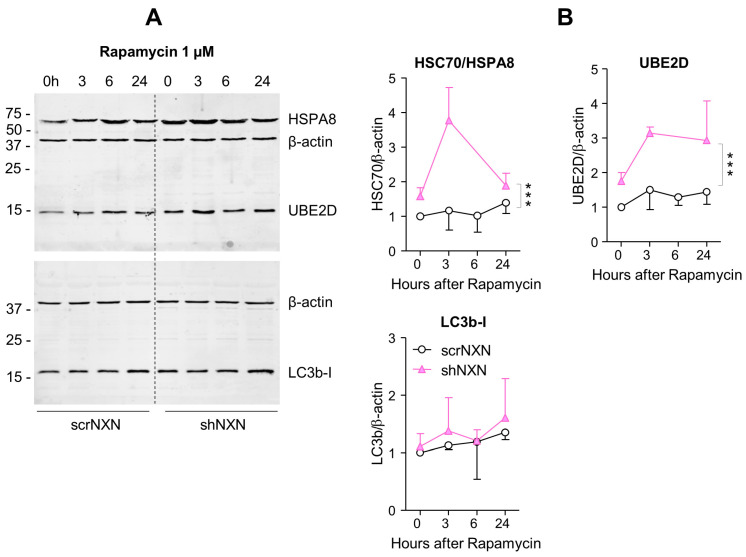
Western blot analysis of autophagy markers upon rapamycin stimulation. (**A**,**B**) In analogy to Figure 3, Western blots show results after stimulation with 1 µM rapamycin. The time courses of HSC70/HSPA8, UBE2D and LC3b were analyzed. Owing to highly stimulated autophagy at this dose, LC3b-II was not detectable. Statistical comparison by Wilcoxon matched pairs rank test, sample size *n* = 3 per group and time point. Asterisks indicate differences between groups *** *p* < 0.001. Raw Western blot data in Supplementary Figure S4.

**Figure 6:**
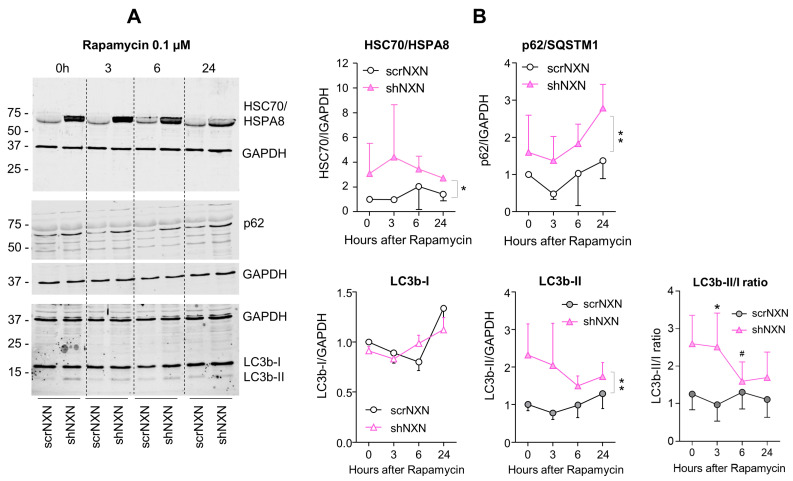
Western blot analysis of autophagy markers upon low-dose rapamycin stimulation. (**A**,**B**) Western blots show the time courses of HSC70/HSPA8, SQSTM1/p62 and LC3b and the LC3b-II/I ratio after stimulation with 0.1 µM rapamycin. Blots were sequentially developed, and infrared images superimposed. Intensities were normalized for loading and show the mean + SD of 3 experiments. Statistical comparison by Wilcoxon matched pairs rank test. Asterisks indicate differences between groups. * *p* < 0.05; ** *p* < 0.01. The # indicates a significant different versus baseline. Raw Western blot data in Supplementary Figures S5 and S6.

Maintenance of high energetic levels requires high rates of autophagy [44,45], which is sensitive to the redox state [13,14,17,46]. We therefore analyzed expression and regulation of redox-sensitive candidates, heat shock proteins HSC70/HSPA8 [13], Hsp90/HSP90A and ubiquitin-2 ligase UBE2D [13] and the general markers of autophagy sequestosome-1 (SQSTM1/p62) and microtubule-associated proteins 1 light chain 3 beta (LC3b-II). Bafilomycin (Figure 4) is an inhibitor of vesicular H^+^-ATPase with an IC50 of 10 nM–0.5 µM, and it prevents lysosomal acidification. It was used at a saturating concentration to completely block autophagosomal degradation so that increases in LC3b-II reveal the rate of autophagosome formation [47]. Rapamycin (Figure 5 and Figure 6) was used to induce mTOR-dependent autophagy, revealing the time-dependent change of LC3b-II and autophagic flux. HSPA8 and HSP90A regulate chaperone-mediated autophagy (CMA) [20,48,49] and are redox-sensitive [13]. The ubiquitin-2 ligase UBE2D acts in concert with HSPA8 in proteasome-mediated removal of oxidized proteins [46]. We therefore opted for these candidates.

Baseline and stimulated expression of HSPA8, HSP90A and UBE2D were increased in shNXN cells, without further upregulation upon stimulation with bafilomycin (Figure 4) but further early and temporary increase in HSPA8 upon a high dose of rapamycin (Figure 5). The permanent stable overall upregulation of these candidates may increase protein turnover capacity.

SQSTM1/p62 and LC3b increased over time upon bafilomycin that blocks the fusion with the lysosome, and hence, results in a block of the autophagic flux with accumulation of autophagosome cargo including p62 and LC3b-II themselves. In shNXN cells, p62 accumulation was stronger, suggesting enhancement of autophagy. LC3b-II also increased but did not reach significance between groups, and the cytosolic LC3b-I remained at baseline. Owing to the rapid fusion with the lysosome, LC3b-II was not detected under high-dose rapamycin, which elicits mTOR-dependent macroautophagy but does not block the flux so that autophagy cargo and LC3b-II are rapidly removed. Again, the cytosolic LC3b-I was unaffected. There was also no consistent difference between groups for LC3b-I and LC3b-II upon stimulation with low dose rapamycin, but in the shNXN cells, LC3b-II/I ratio dropped significantly from a higher baseline level, suggesting a higher autophagic flux under this stimulation.

From the autophagy experiments, we infer that NXN knockdown enhances autophagy and CMA without an effect on autophagolysosomal fusion or lysosomal degradation. High overall expression of HSP70, HSP90 and UBE2D may be interpreted as adaptive mechanisms to NXN deficiency to maintain high proliferation and functioning irrespective of a loss of redox regulation.

## 4. Discussion

We show in the present study that knockdown of NXN increases renewal and proliferation of SH-SY5Y human neuroblastoma cells in association with higher energy consumption and persistent upregulation of redox-sensitive chaperones and UBE2D. SY-SY5Y cells are a model for neuronal aging and neurodegenerative diseases [50], and we have recently shown that oxidation of HSC70/HSPA8 and UBE2D leads to premature SH-SY5Y cell senescence [13]. Hence, the phenotype of shNXN cells with high cell cycle reentry, high energetic level and high rate of stimulated autophagy suggests that NXN knockdown in neuronal cells favors rejuvenation. Considering that NXN is supposed to maintain WNT signaling [8], a key pathway for development and cell differentiation [6,51,52] but also growth in cancer [53,54], one would rather expect that NXN knockdown stops proliferation. However, WNT signaling is context-sensitive [55] and drives maturation in SH-SY5Y cells [56]. Hence, the pro-proliferating phenotype of shNXN cells does not contradict the notion that NXN promotes WNT signaling.

NXN is an oxidoreductase, and the net redox outcome for susceptible proteins likely depends on the experimental conditions and cell type. A recent redox proteomic study in SH-SY5Y cells revealed that NXN deletion mostly resulted in a higher fraction of reduced proteins, suggesting that NXN acted primarily as oxidase for these candidates, which were mainly involved in development and cell morphology [7]. In combination with our studies, one may hypothesize that NXN-mediated oxidation drives differentiation. We have not specifically addressed redox-mediated protein alterations of NXN in our study. We were primarily interested in overall consequences of the knockdown, which are not necessarily a direct and exclusive function of its oxidoreductase activity. Indeed, the observed upregulations of HSPA8, HSP90A and UBE2D, which are likely part of a more complex adaptive response towards knockdown of NXN, suggest an active role in transcriptional regulation and proteostasis. Proteasome- and autophagy-mediated breakdown of disheveled and -catenin counteract WNT signaling, providing thereby a negative feedback regulation [53,54]. NXN has not yet been studied in the context of autophagy or autophagy flux, but a previous interaction screen found HDAC6 as a putative interactor [16]. HDAC6 controls ubiquitin-mediated [57] and chaperone-mediated autophagy [58].

Owing to embryonic lethality and dysmorphic features of homozygous conventional NXN knockout mice (www.mousephenotype.org, accessed on 5 February 2021, MGI:109331), we had expected that NXN knockdown in SH-SY5Y cells would impair their viability. Instead, proliferation and cell cycle reentry were increased in shNXN cells, suggesting that NXN drives quiescence in neurons, and that adaptations to its loss allow for high energy turn over and drive renewal. The knockdown efficacy at the immunofluorescence level was near complete, but at the Western blot level, about 20–25% expression of NXN remained, suggesting efficient knockdown but not complete loss. Our data suggest that the adaptation to the knockdown was mediated at least in part by upregulation of the chaperones and UBE2D and likely enhancement of autophagy. The experiments with bafilomycin, which blocks lysosomal acidification and hence, cargo breakdown including LC3b-II itself, had suggested an increase in autophagolysosome formation in shNXN cells. In addition, low dose rapamycin experiments that stimulates mTOR dependent autophagy suggested a higher autophagic flux because LC3b-II significantly dropped in shNXN cells under this stimulation, albeit from a higher baseline level in this experiment. Autophagy is a prerequisite for cell renewal of stem cells [44,59,60,61] and neurogenesis from neural stem cells throughout adult life [62]. Since SH-SY5Y cells are a model of neuronal aging [13] and neurodegenerative diseases [50,63], one may conclude that NXN downregulation, antagonism or pharmacologic inhibition might be able to combat aging, if it were to occur in vivo.

Redoxins have hardly been studied in autophagy. Thus far, only peroxiredoxin-2 was reported to promote MAP kinase-driven autophagy, fostering thereby cancer cell proliferation [64]. The literature exclusively reports that knockout or knockdown of a redoxin, either a peroxiredoxin or a thioredoxin, inhibits cancer cell proliferation [64,65,66,67] and that overexpression protects against various types of cellular stressors [68,69,70,71] and promotes cancer cell growth or drug resistance [72,73,74]. There might be a certain bias in reporting expected redoxin effects, or NXN is special in that it rather sustains protein oxidation [7], or has functions aside its redox capabilities.

## 5. Conclusions

We conclude that the redox challenges brought about by NXN knockdown lead to permanent upregulations of redox-sensitive candidate proteins, which are involved in autophagic protein degradation. As a result, shNXN SH-SY5Y cells show a stronger autophagosome formation under stimulation, a higher rate of cell renewal, and enhanced basal cell respiration. In neuronal cells, enhancement of autophagy and maintenance of high neurogenesis are proposed as anti-aging strategies, best achieved with dietary restriction and physical exercise. SH-SY5Y cells are a model of aging, senescence and neurodegenerative diseases. Our data suggest that NXN inhibition might help to achieve a pro-renewal state if such features were to be met with NXN inhibitors in vivo. This might be a valuable anti-aging strategy but might foster cancer growth.

## Data Availability

The data generated for this manuscript are presented within the manuscript or in Supplementary Figures. Raw data shall be made available on reasonable request.

## Supplementary Materials

The following are available online at https://www.mdpi.com/2076-3921/10/3/449/s1. Supplementary Table S1 shows the antibodies. Supplementary Figures show exemplary cell cycle histograms (Figure S1), and raw uncut Western Blots (S2, S3, S4, S5 and S6). Figure S1: Cell cycle analysis of naive and H2O2-treated shNXN SH-SY5Y neuroblastoma cells versus scrambled control cells (scrNXN); Figure S2: Uncut raw Western Blots presented in Figure 4, part 1; Figure S3: Uncut raw Western Blots presented in Figure 4, part 2; Figure S4: Uncut raw Western Blots presented in Figure 5; Figure S5: Uncut raw Western Blots presented in Figure 6, part 1; Figure S6: Uncut raw Western Blots presented in Figure 6, part 2.
