# Supplementary material for: Nucleoredoxin Knockdown in SH-SY5Y Cells Promotes Cell Renewal
Source: Antioxidants (Basel). 2021 Mar 13;10(3):449. doi: 10.3390/antiox10030449 (PMC7999887; doi:10.3390/antiox10030449)
Supplement: Supplementary file 1 [file antioxidants-10-00449-s001.zip › NXN-sushi_Suppl.Table_SupplFigures_2020-02-27.pdf]

Suppl. Table 1: Antibodies

Antibody	Seller	Product #	Dilution	Host	Type	Protocol	Use
**β-actin**	Sigma	A5060	1:1000	rabbit	pab	1 h, RT	WB
**Cytochrome oxidase COX-IV**	Abcam	ab33985	1:100	mouse	mab	1 d, 4°C	IF
**GAPDH**	Ambion	AM4300	1:1000	mouse	mab	1 d, 4°C	WB
**Hsc70/HSPA8**	Abcam	ab19136	1:200	rat	mab	1 d, 4°C	WB
**Ki67**	Abcam	ab15580	1:500	rabbit	pab	1 d, 4°C	IF
**LC3b**	Cell Signaling	2775S	1:300<br>1:200	rabbit	pab	3 d, 4°C	IF<br>WB
**Nucleoredoxin**	Sigma	HPA023566	1:200	rabbit	pab	1 d, 4°C	IF, WB
**SQSTM1/p62**	Abcam	ab155686	1:200	rabbit	pab	3 d, 4°C	WB
**Ubiquitin C (UBC)**	Cell Signaling	3936	1:200	mouse	mab	3 d, 4°C	WB
**UBE2D2**	Santa Cruz	sc166278	1:200	mouse	mab	3 d, 4°C	WB
**Vimentin**	Abcam	ab92547	1:100	rabbit	mab	1 d, 4°C	IF
**Drp1**	Cell Signal	D6C7 #8570	1:200	rabbit	mab	1 d, 4°C	IF
**Wheat germ agglutinin, AF488 conjugate**	Invitrogen	W11261	5 µg/ml			10 min, 37°C	IF

WB, Western Blot; IF, immunofluorescence

Suppl. Fig. 1

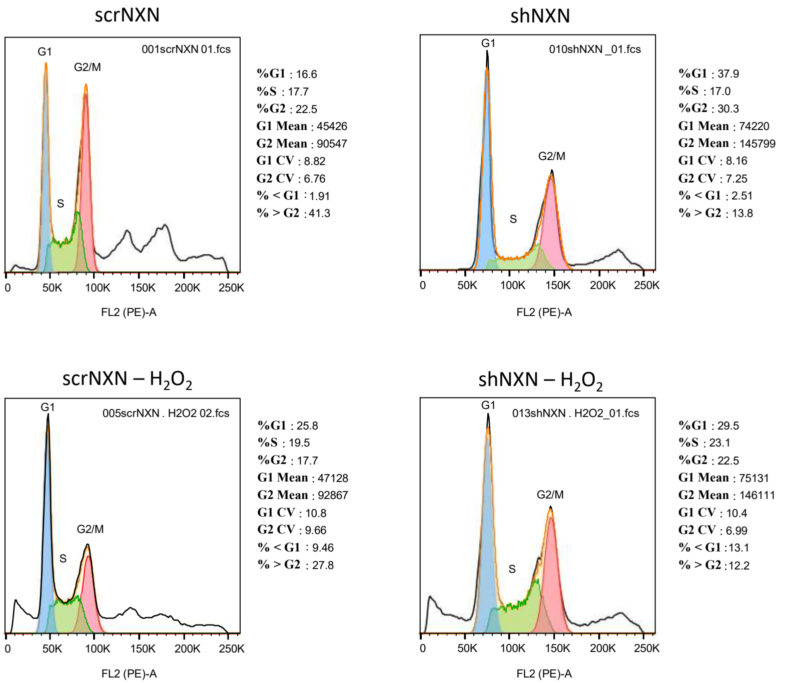

Suppl. Fig. 1: Cell cycle analysis of naive and H_2O_2-treated shNXN SH-SY5Y neuroblastoma cells versus scrambled control cells (scrNXN). The cell cycle distribution was fitted to an univariate algorithm similar to a Watson Pragmatic algorithm with FlowJo V10.6. The images show exemplary results for each group.

Suppl. Fig. 2: Bafilomycin 1 µM (blots of Fig. 4)

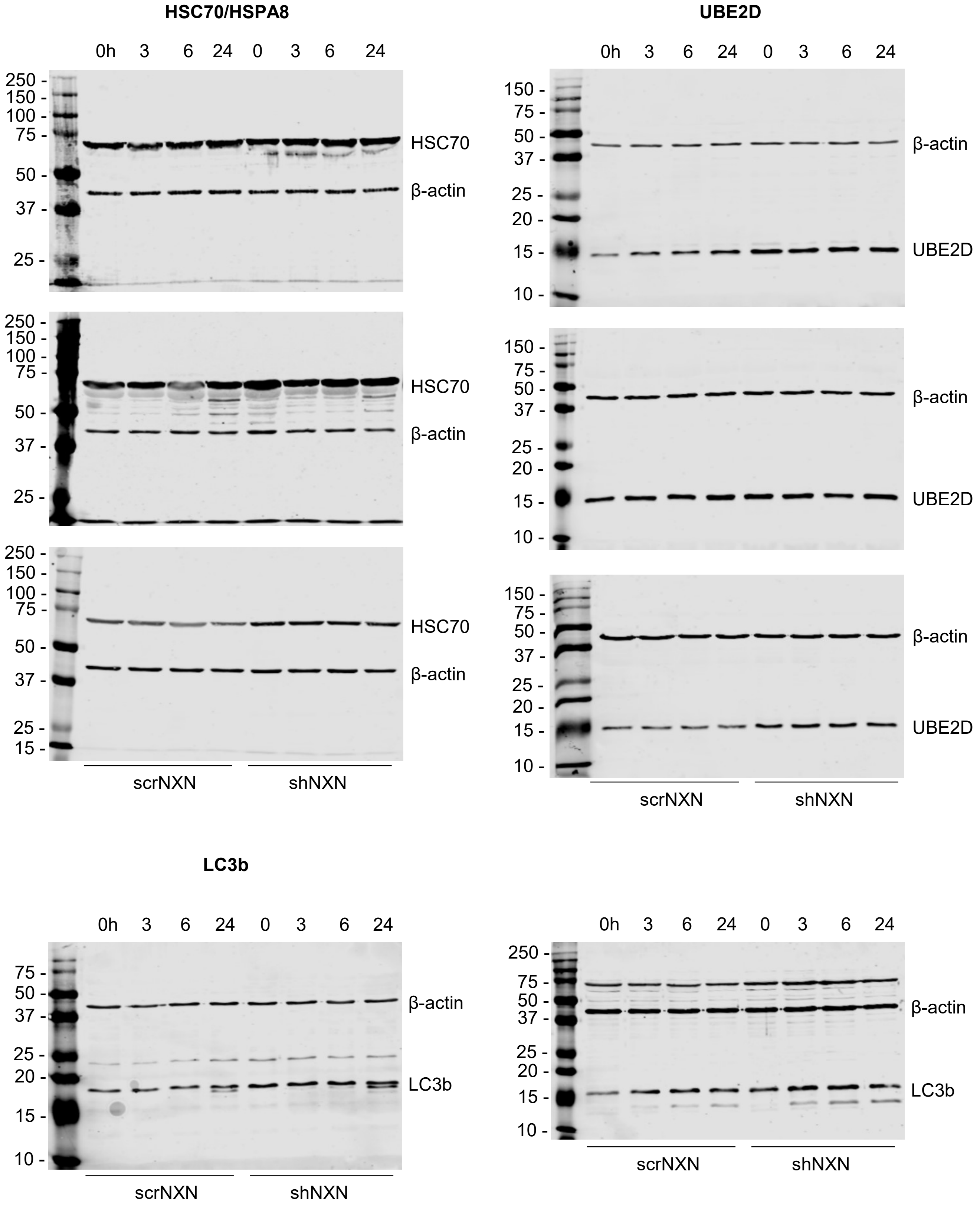

Suppl. Fig. 2: Uncut raw Western Blots presented in Figure 4. shNXN, shRNA against NXN, stably tranduced SH-SY5Y neuroblastoma cells; scrNXN, scrambled shRNA transduced control SH-SY5Y cells. Blots were sequentially developed and analyzed using infrared technology

Suppl. Fig. 3 Bafilomycin 1 µM (blots of Fig. 4)

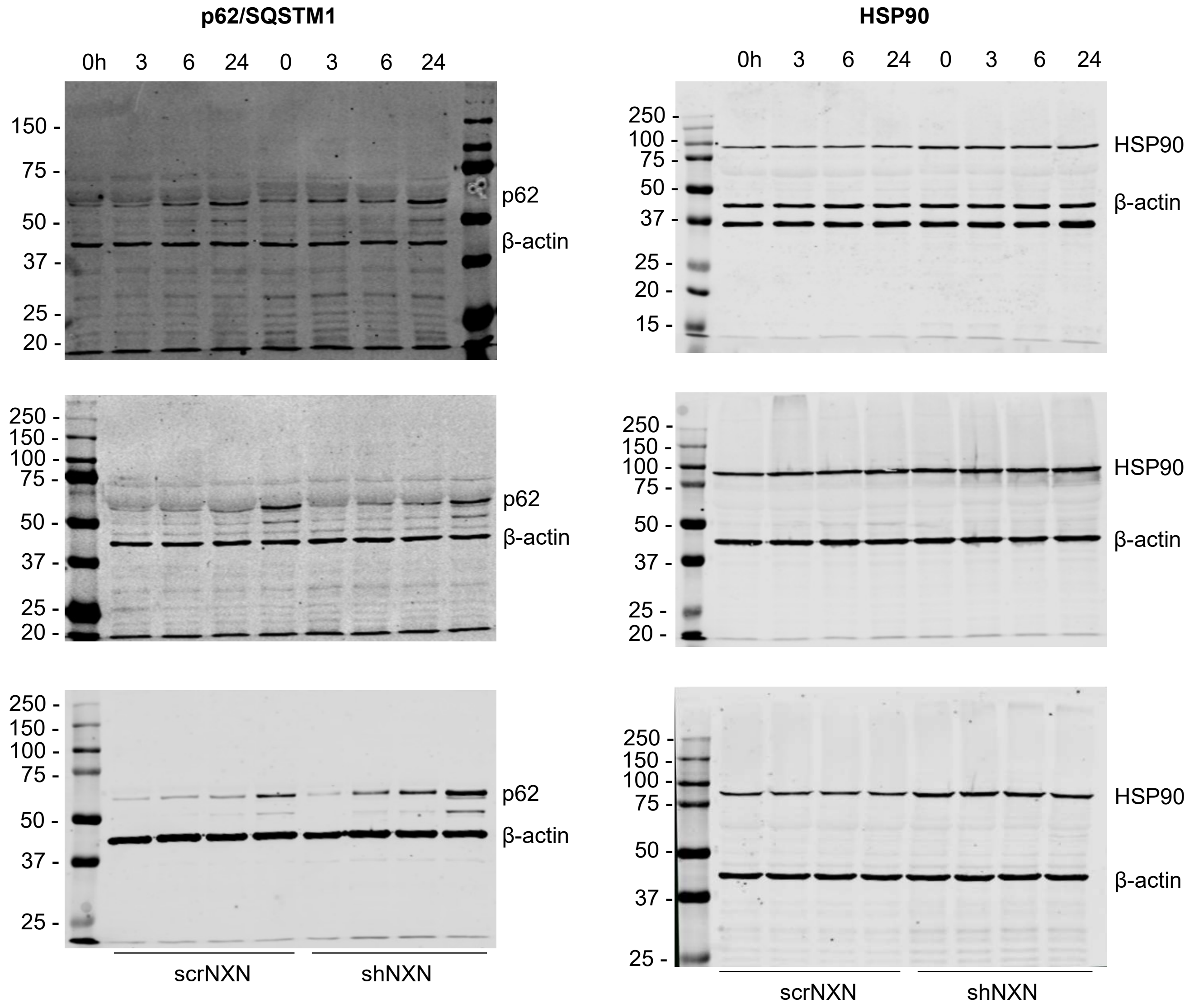

Suppl. Fig. 3: Uncut raw Western Blots presented in Figure 4. shNXN, shRNA against NXN, stably tranduced SH-SY5Y neuroblastoma cells; scrNXN, scrambled shRNA transduced control SH-SY5Y cells. Blots were sequentially developed and analyzed using infrared technology

Suppl. Fig. 4 Rapamycin 1 µM (blots of Fig. 5)

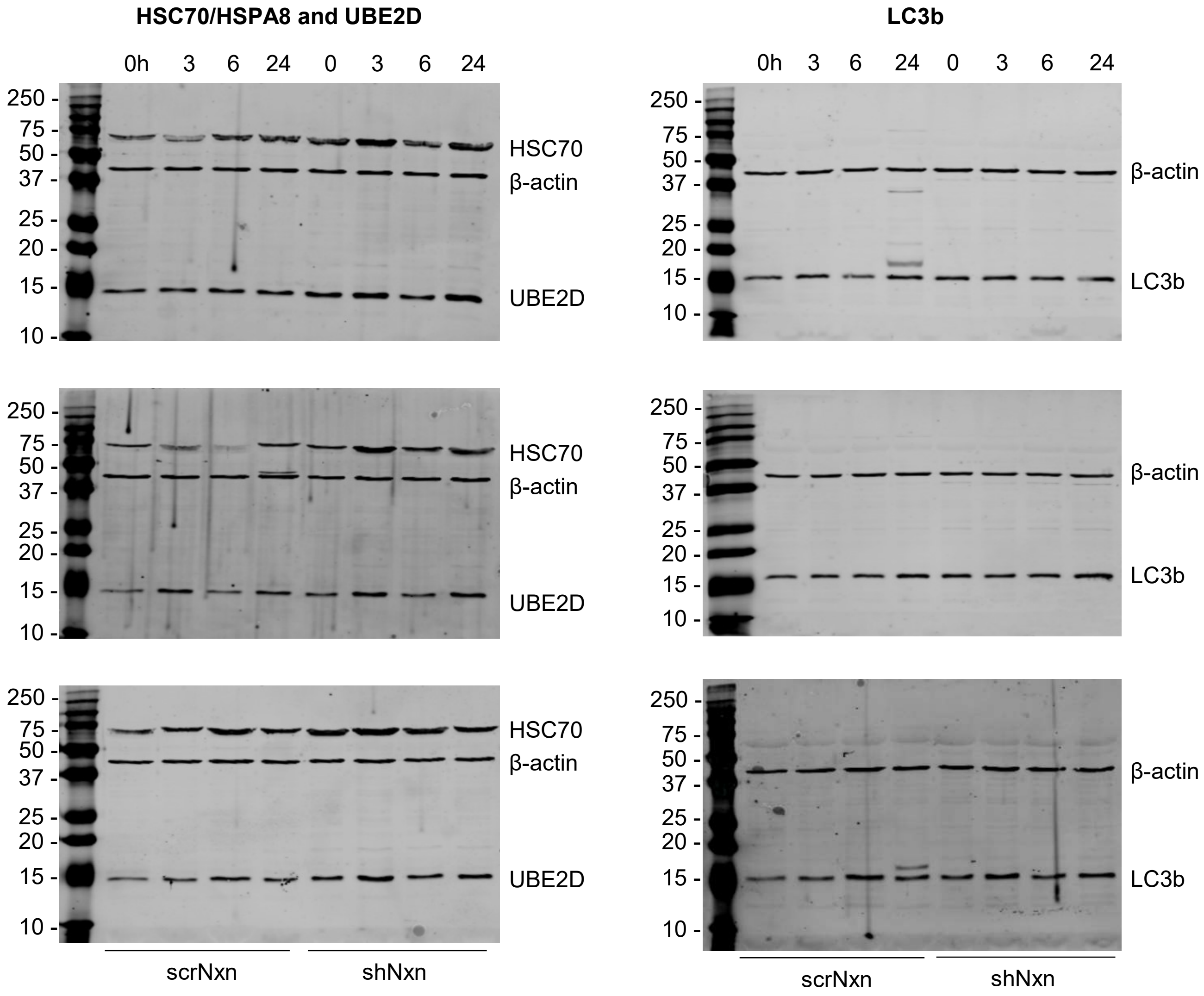

Suppl. Fig. 4: Uncut raw Western Blots presented in Figure 5. shNXN, shRNA against NXN, stably tranduced SH-SY5Y neuroblastoma cells; scrNXN, scrambled shRNA transduced control SH-SY5Y cells. Blots were sequentially developed and analyzed using infrared technology

Suppl. Fig. 5 Rapamycin 0.1 µM (blots of Fig. 6)

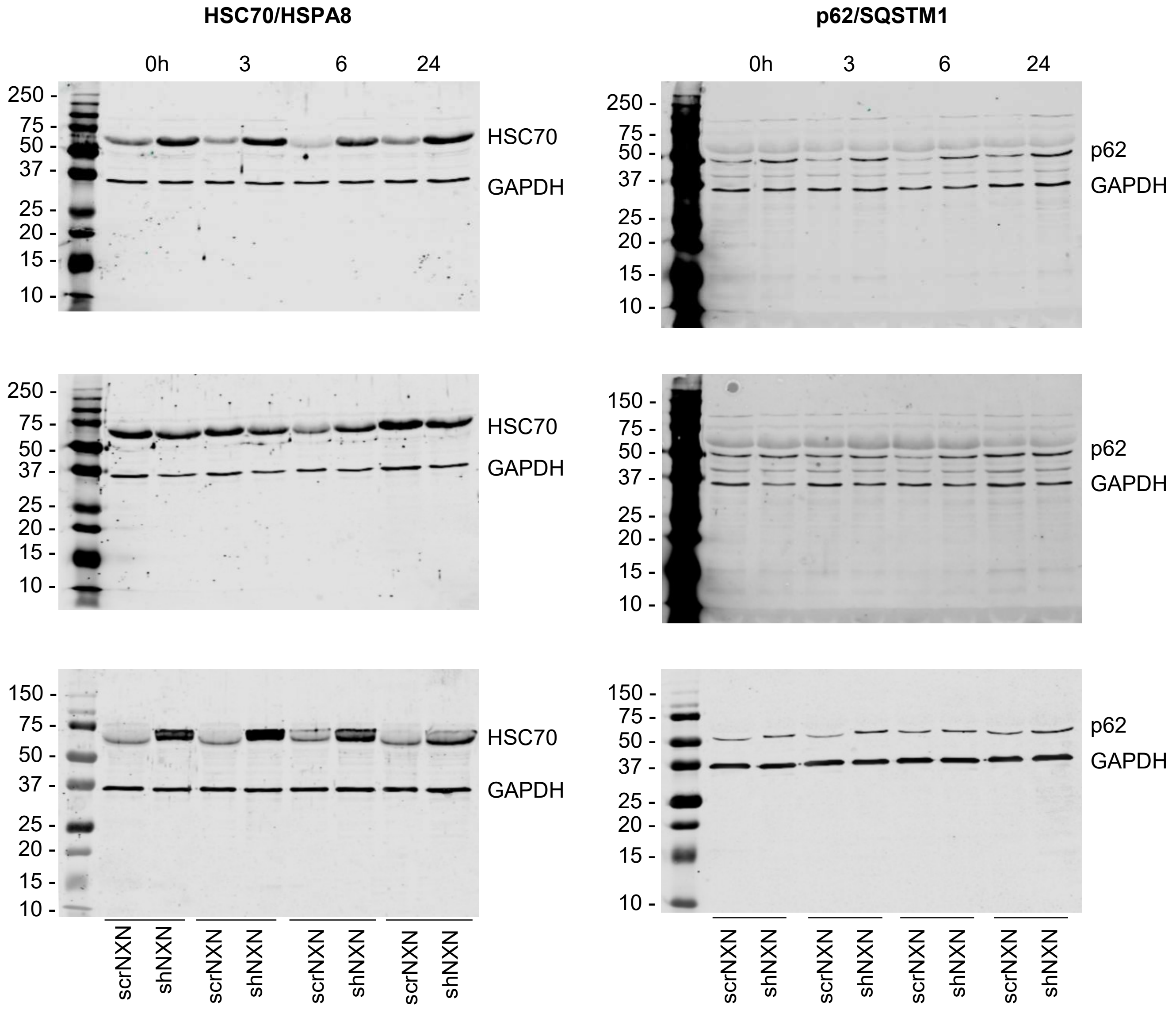

Suppl. Fig. 5: Uncut raw Western Blots presented in Figure 6. shNXN, shRNA against NXN, stably tranduced SH-SY5Y neuroblastoma cells; scrNXN, scrambled shRNA transduced control SH-SY5Y cells. Blots were sequentially developed and analyzed using infrared technology

Suppl. Fig. 6 Rapamycin 0.1 µM (blots of Fig. 6)

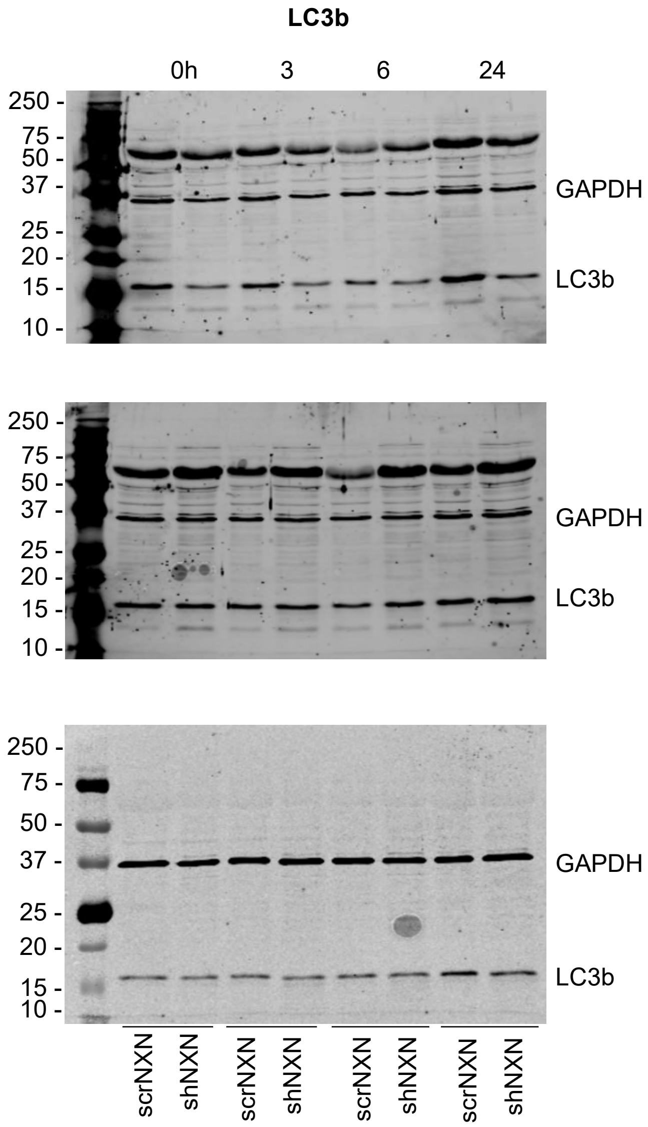

Suppl. Fig. 6: Uncut raw Western Blots presented in Figure 6. shNXN, shRNA against NXN, stably tranduced SH-SY5Y neuroblastoma cells; scrNXN, scrambled shRNA transduced control SH-SY5Y cells. Blots were sequentially developed and analyzed using infrared technology
