# Supplementary figures and images for: Nucleoredoxin Knockdown in SH-SY5Y Cells Promotes Cell Renewal
Source: Antioxidants (Basel). 2021 Mar 13;10(3):449. doi: 10.3390/antiox10030449 (PMC7999887; doi:10.3390/antiox10030449)

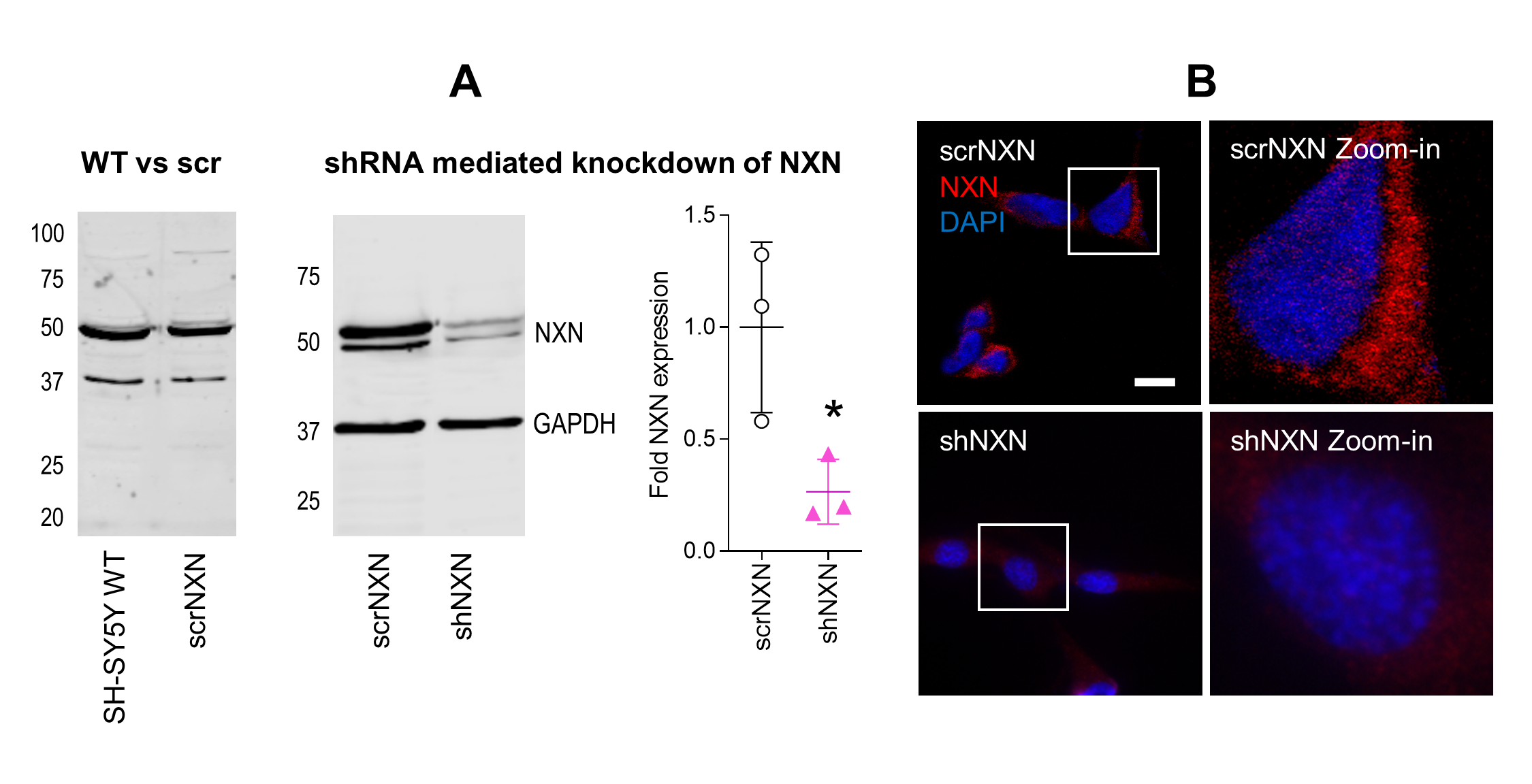

Supplement: Supplementary file 1 [file antioxidants-10-00449-s001.zip › Fig1.tif]

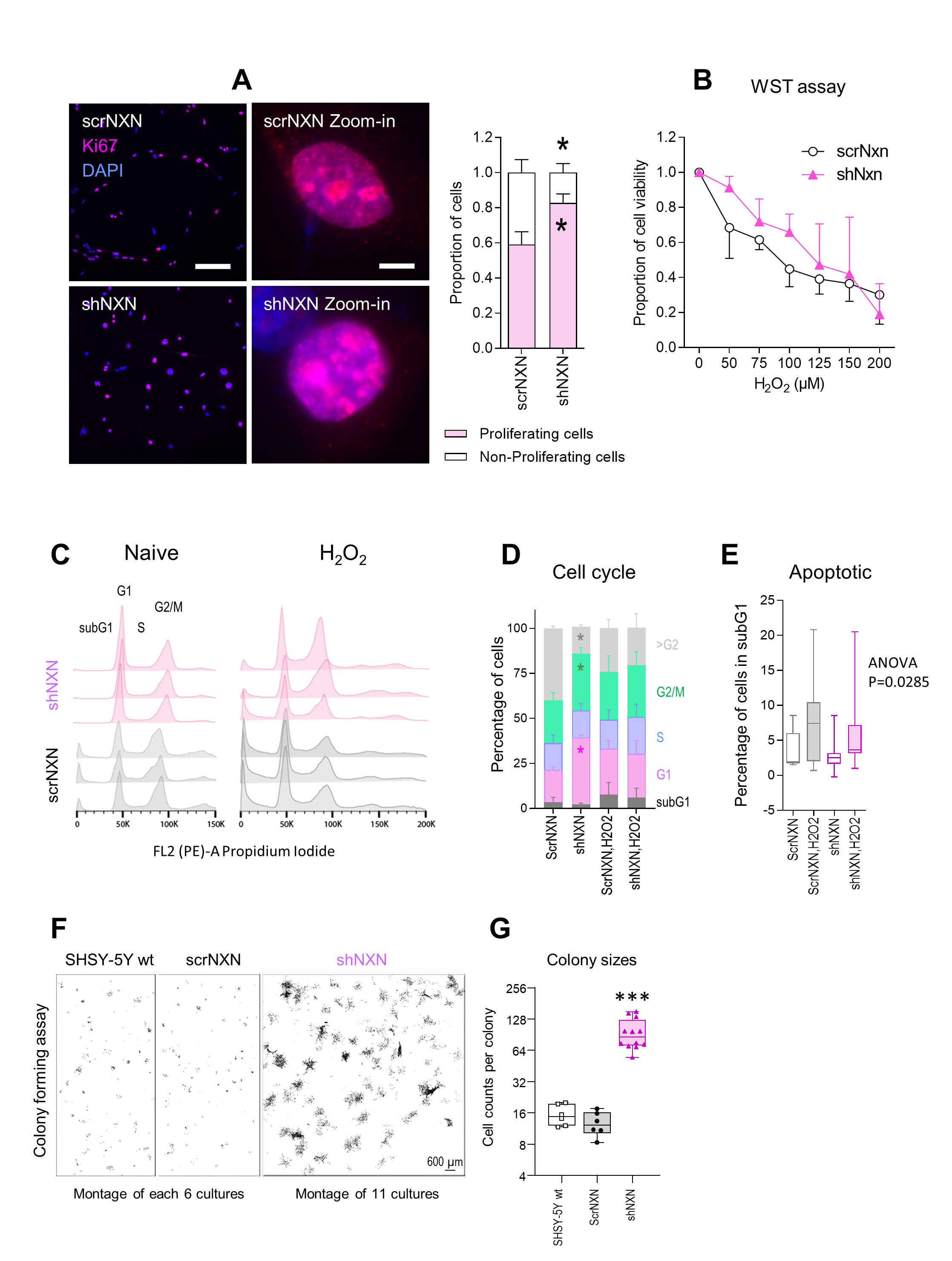

Supplement: Supplementary file 1 [file antioxidants-10-00449-s001.zip › Fig2.tif]

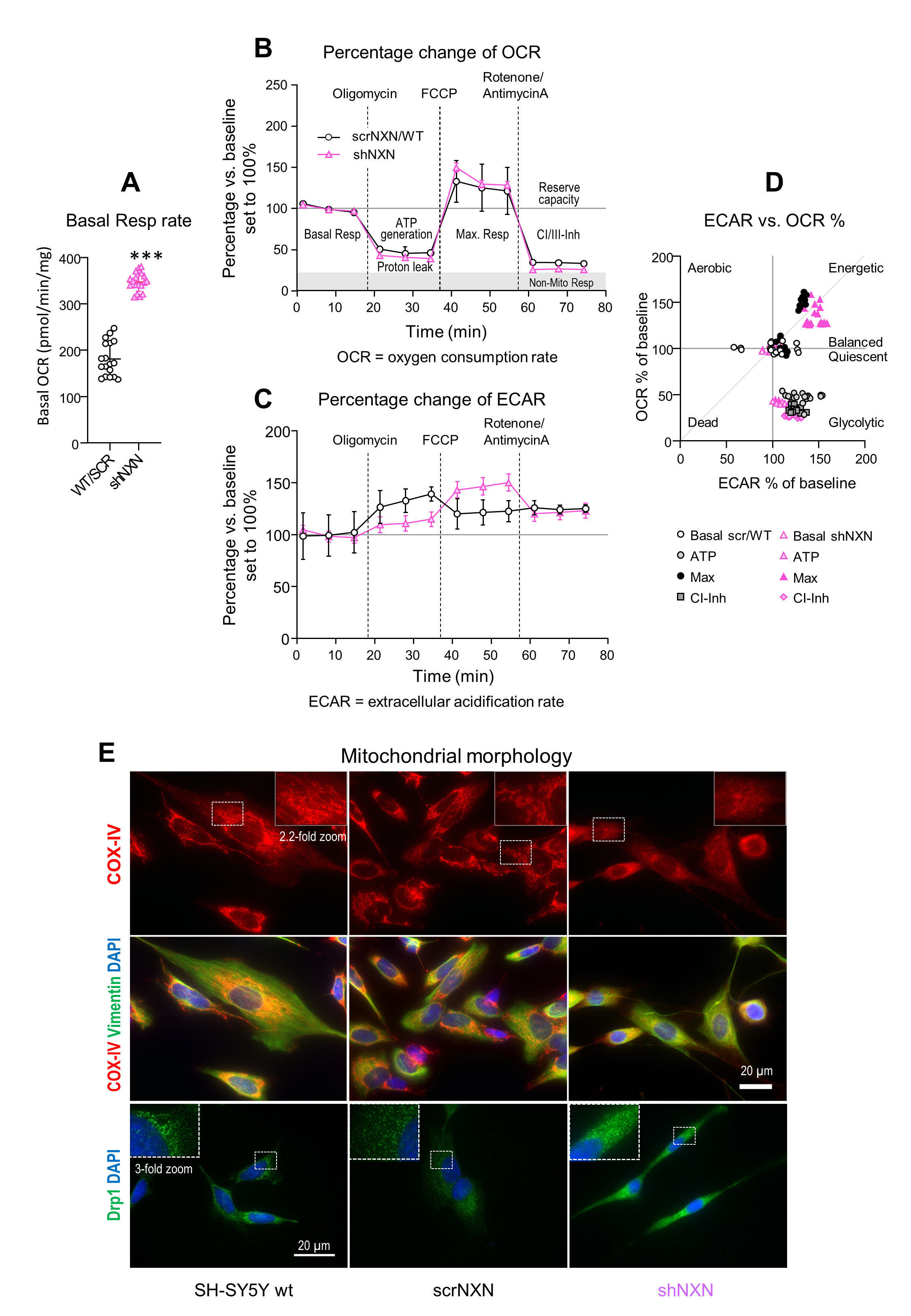

Supplement: Supplementary file 1 [file antioxidants-10-00449-s001.zip › Fig3_R2.tif]

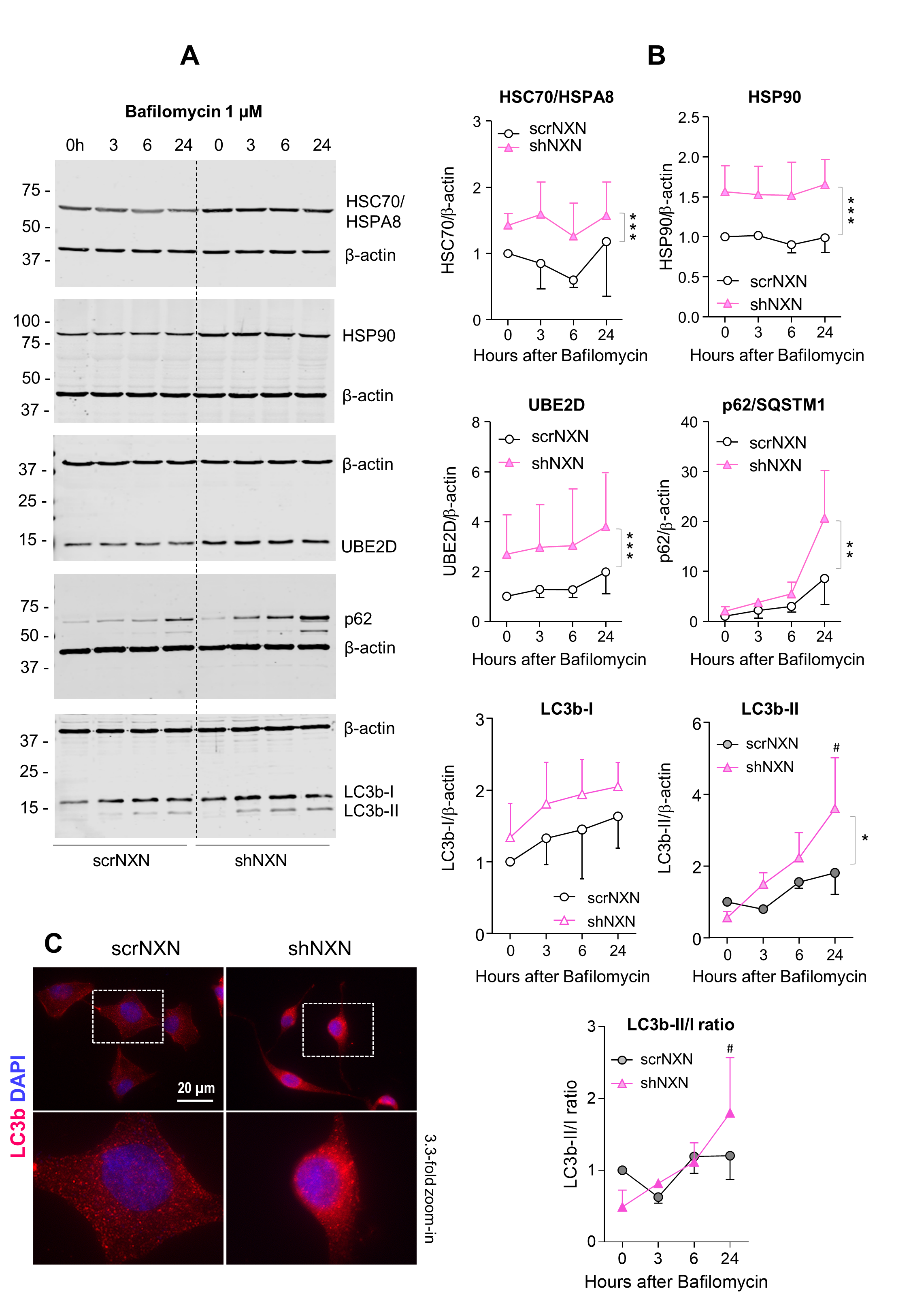

Supplement: Supplementary file 1 [file antioxidants-10-00449-s001.zip › Fig4_R2.tif]

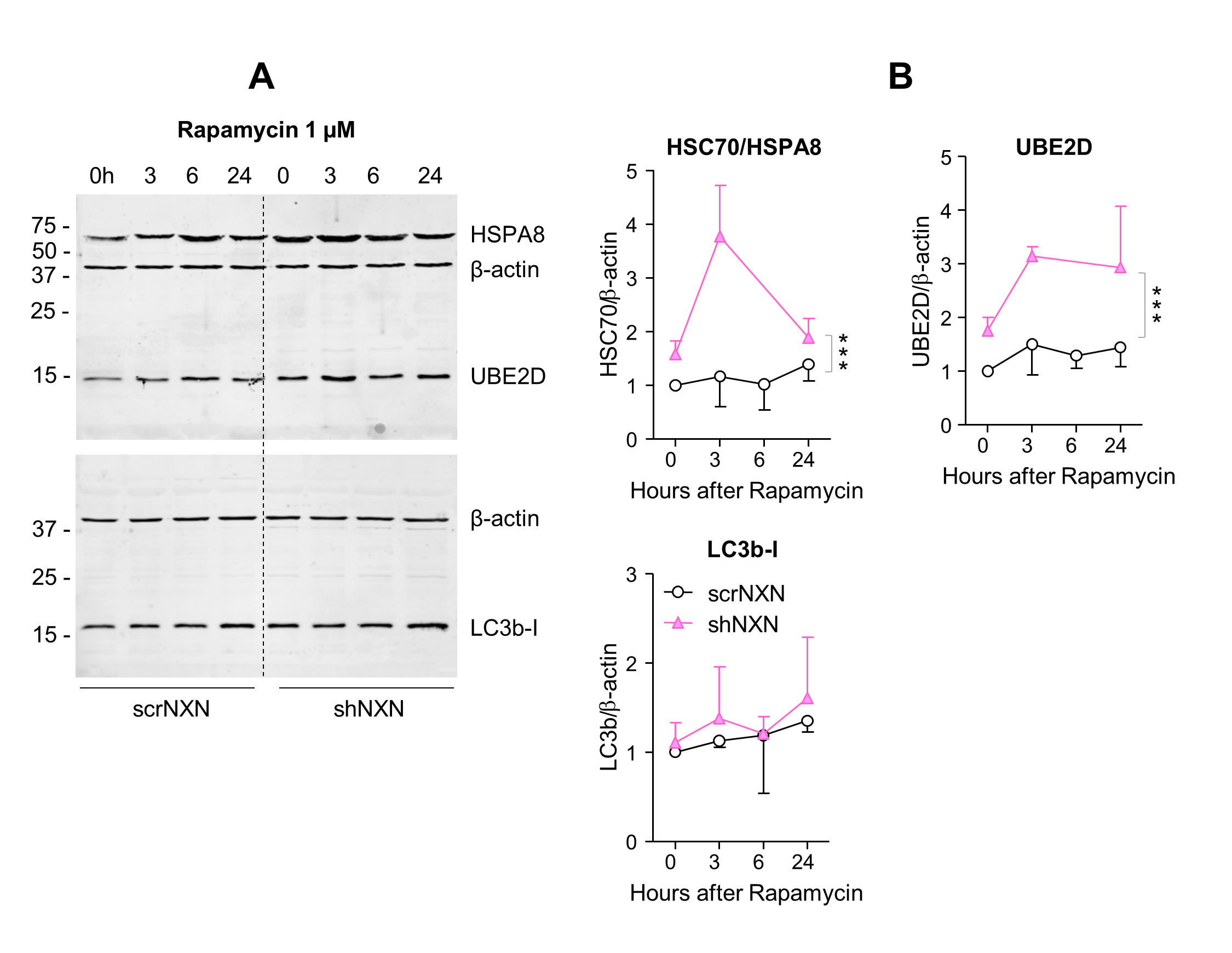

Supplement: Supplementary file 1 [file antioxidants-10-00449-s001.zip › Fig5.tif]

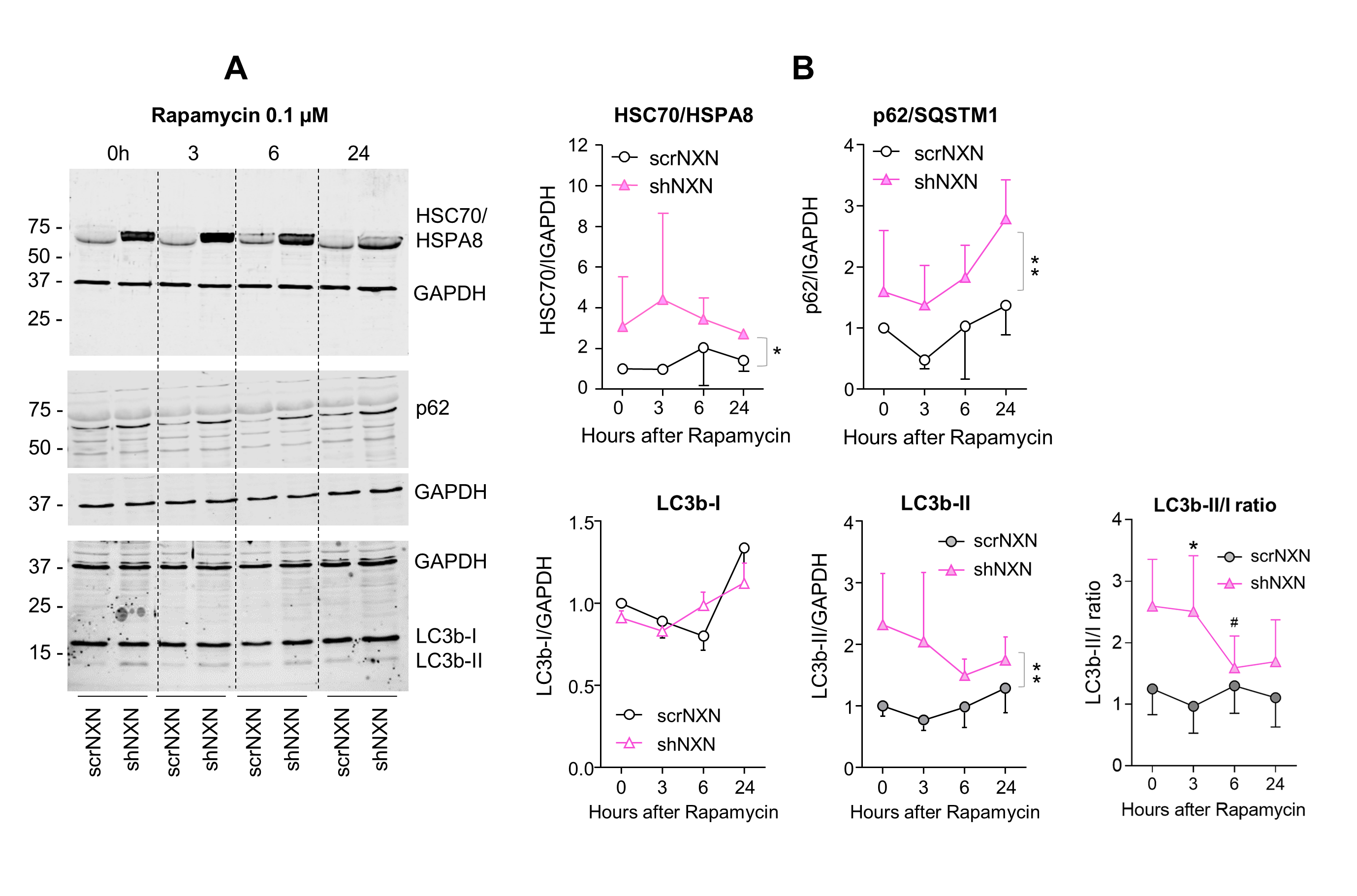

Supplement: Supplementary file 1 [file antioxidants-10-00449-s001.zip › Fig6-quer.tif]
